# Clues toward precision medicine in oral squamous cell carcinoma: utility of next-generation sequencing for the prognostic stratification of high-risk patients harboring neck lymph node extracapsular extension

**DOI:** 10.18632/oncotarget.11762

**Published:** 2016-08-31

**Authors:** Hung-Ming Wang, Chun-Ta Liao, Tzu-Chen Yen, Shu-Jen Chen, Li-Yu Lee, Chia-Hsun Hsieh, Chien-Yu Lin, Shu-Hang Ng

**Affiliations:** ^1^ Department of Internal Medicine, Division of Medical Oncology, Chang Gung Memorial Hospital and Chang Gung University, Taoyuan, Taiwan, ROC; ^2^ Department of Otorhinolaryngology, Section of Head and Neck Surgery, Chang Gung Memorial Hospital and Chang Gung University, Taoyuan, Taiwan, ROC; ^3^ Department of Nuclear Medicine and Molecular Imaging Center, Chang Gung Memorial Hospital and Chang Gung University, Taoyuan, Taiwan, ROC; ^4^ ACT Genomics, Taipei, Taiwan, ROC; ^5^ Department of Pathology, Chang Gung Memorial Hospital and Chang Gung University, Taoyuan, Taiwan, ROC; ^6^ Department of Radiation Oncology, Chang Gung Memorial Hospital and Chang Gung University, Taoyuan, Taiwan, ROC; ^7^ Department of Diagnostic Radiology, Chang Gung Memorial Hospital and Chang Gung University, Taoyuan, Taiwan, ROC

**Keywords:** next-generation sequencing, precision medicine, oral squamous cell carcinoma, extracapsular extension, TP53 DNA-binding domain

## Abstract

Patients with resected oral squamous cell carcinoma (OSCC) harboring extracapsular extension (ECE) of the involved lymph node, show poor and heterogeneous outcomes. We aim to improve their prognostic stratification by combining genetic information from next-generation sequencing (NGS) using traditional clinicopathological prognosticators. The hotspot mutation regions of 45 cancer-related genes were investigated using NGS with an ultra-deep (>1000×) sequencing approach in formalin-fixed paraffin-embedded samples obtained from 201 patients with resected OSCC harboring ECE. Adjuvant chemoradiotherapy (CRT) and the number of nodes with ECE were the most important traditional prognosticators for disease-specific survival (DSS). The 5-year DSS for patients with CRT *versus* without, was 55% *versus* 21% (*P* < 0.001), and that for 1-3 *versus* ≥ 4 ECEs was 60% *versus* 25% (*P* = 0.001), respectively. Multivariate analysis in patients who received adjuvant CRT for 1-3 ECEs (i.e., those with a favorable expected prognosis) identified the following adverse prognostic factors: 1) margin of < 5 mm for locoregional failure (66% *versus* 30%, *P* = 0.007) and DSS (42% *versus* 63%, *P* = 0.039); 2) HRAS mutation for distant failure (55% *versus* 25%, *P* = 0.007) and DSS (36% *versus* 63%, *P* = 0.024); and 3) TP53 DNA-binding domain missense mutations for DSS (52% *versus* 71%, *P* = 0.025) and overall survival (39% *versus* 61%, *P* = 0.007).

We conclude that genetic information from NGS may improve the prognostic stratification offered by traditional prognosticators in resected OSCC patients with ECE. Our findings will contribute to implementation of precision medicine in OSCC patients.

## INTRODUCTION

Oral malignancies pose a significant health burden and frequently require complex and expensive treatment. In particular, head and neck squamous cell carcinoma (HNSCC) is the fifth-most common malignancies worldwide, with an incidence of approximately 650,000 new cases per year [1]. Moreover, it ranks as the fourth-leading cause of cancer-related death in Taiwanese males [2]. Owing to the endemic habit of chewing betel quid in Southern Asia, oral squamous cell carcinoma (OSCC) accounts for nearly 70% of all new HNSCC cases in Taiwan [2].

Upfront surgery continues to remain the mainstay of treatment for patients with resectable OSCC. The adverse prognostic impact of extracapsular extension (ECE) in OSCC has been consistently demonstrated [3–6]. A meta-analysis of nine studies collectively examining 2573 patients with HNSCC has shown that the risk of death is 2.7-fold higher when ECE is present in neck lymph nodes [7]. Post-operative, cisplatin-based, adjuvant CRT is currently considered the standard of care for patients with pathologically proven positive margins and/or ECE [8–10]. However, the relatively high locoregional failure (LRF) (22.3%) and poor disease-free survival (DFS) rates (20.1%) in the long-term [11] pose major clinical hurdles. On the other hand, approximately 40% of patients with ECE achieve a 5-year disease-specific survival (DSS) of 66% [6]. In this scenario, improved stratification approaches for high-risk patients with resected HNSCC are eagerly awaited. Histopathological factors may refine the prediction of outcomes in OSCC [4, 6], and molecular profiling holds great promise for predicting prognosis and devising tailored treatment approaches.

We have previously studied the hotspot mutation regions of 45 cancer-related genes with an ultra-deep (>1000×) sequencing approach, using next-generation sequencing (NGS) of formalin-fixed paraffin-embedded (FFPE) samples obtained from 345 patients with resected OSCC [12]. The frequency of genetic variations in tumor suppressor genes identified in our study was largely similar to that observed in The Cancer Genome Atlas (TCGA) HNSCC dataset (containing whole-exome sequencing data of 279 tumors) [13]. However, we found a lesser degree of sequence variations for the CDKN2A (12.8% *versus* 22.6% in the TCGA data) and NOTCH1 (3.2% *versus* 18.6% in the TCGA data) genes. In contrast, several oncogenes (including potential drug targets) showed a 3-fold higher mutation rate in our sample than that in the TCGA dataset. Differences in exposure to known oral carcinogens, disease stage, ethnicity, or genetic background may at least in part explain such discrepancies. Importantly, we previously identified a genetic signature that independently predicted a poorer DFS [12]. Subsequently, in a previous study focussing on TP53, the most commonly mutated gene in HNSCC, we correlated the value of missense mutations affecting TP53 DNA-binding domain (DBD) (but not of the remaining TP53 mutations) with DSS [14].

In recent years, the paradigm of precision medicine in patients with malignancies has been gaining momentum. In the field of OSCC, the goal is to personalize prognostication and treatment strategies as a function of patient-specific somatic mutations and aberrant molecular pathways. In this scenario, we conducted a substudy of our original cohort subjected to NGS [12] by specifically focusing on patients with ECE (*n* = 201). Our main goal was to improve the prognostic stratification of high-risk patients by combining genetic information from NGS with traditional clinicopathological prognostic parameters.

## RESULTS

### General characteristics of patients with and without ECE

Between 1996 and 2011, we identified 345 OSCC patients. The median follow-up duration was 41.0 months (range: 1−214 months). In the subgroup of patients with ECE (*n* = 201), the median follow-up duration was 24.0 months (range: 0−179 months). The general characteristics of patients with and without ECE are shown in Table 1 and Supplementary Table S1 (genes with a mutation rate of < 2% patients). Compared with patients without ECE, those with ECE showed a higher frequency of T3-4, N2b-c, Stage IV, poor differentiation, near margin, perineural and lymphatic invasion, and level 4-5 lymph node involvement. They also showed a deeper tumor invasion. The frequency of patients harboring genetic mutations in patients with and without ECE was 73.6% and 69.4%, respectively (*P* = 0.394), with the mean number of mutations being 1.37 ± 2.35 and 1.01 ± 1.13, respectively (*P* = 0.056).

**Table 1:** General characteristics of the study patients stratified according to the presence of neck lymph node extracapsular extension

	ECE (−)		ECE (+)				ECE (−)		ECE (+)		
	n = 144		n = 201				n = 144		n = 201		
Clinical variables	n	%	n	%	*P* value	Clinical variables	n	%	n	%	***P*** value
Sex					0.440	Margin < 5 mm	11	7.6	32	16.0	0.021
Male	134	93.1	191	95		Depth > 10 mm	88	61.1	143	71.1	0.051
Female	10	6.9	10	5		Tumor invasion					
Age, years					0.761	Bone	27	18.8	44	21.9	0.477
Mean	49.4 ± 10.5		49.8 ± 11.3			Skin	12	8.3	25	12.4	0.224
Age >70 years	8	5.6	8	4	0.605	Perineural	64	44.4	113	56.2	0.031
Cancer site					0.552	Vascular	6	4.2	12	6.0	0.458
Tongue	56	38.9	74	36.8		Lymphatic	12	7.6	33	16.4	0.016
Mouth floor	6	4.2	9	4.5		Level 4/5 lymph nodes	4	2.8	23	11.4	0.004
Lip	1	0.7	1	0.5		HPV					0.526
Buccal	52	36.1	80	39.8		None	115	88.5	159	85.0	
Gum	17	11.8	27	13.4		Type 16, 18	15	11.5	27	14.4	
Hard palate	5	3.5	1	0.5		Other type^a^	0	0	1b	0.5	
Retromolar	7	4.9	9	4.5		Genetic variables	n	%	n	%	*P* value
Tumor status					0.084	Presence of mutations	100	69.4	148	73.6	0.394
1	10	6.9	6	3.0		Number of mutations (mean)	1.01 ± 1.13		1.37 ± 2.35		0.056
2	64	44.4	73	36.3		TP53	78	54.2	123	61.2	0.192
3	27	18.8	43	21.4		TP53 DBD missense mutations (n = 333)	59	42.1	98	50.8	0.119
4	43	29.9	79	39.3							
Lymph node status					< 0.001	CDKN2A	15	10.4	23	11.4	0.764
N1	91	63.2	32	15.9		PIK3CA	12	8.3	23	11.4	0.346
N2a	0	0	3	1.5		HRAS	9	6.3	20	10.0	0.222
N2b	48	33.3	138	68.7		BRAF	4	2.8	7	3.5	0.768
N2c	5	3.5	28	13.9		EGFR	4	2.8	6	3.0	1.000
Stage					< 0.001	FGFR3	1	0.7	6	3.0	0.246
III	63	43.8	22	10.9		SMAD4	1	0.7	6	3.0	0.246
IV	81	56.3	179	89.1		KDR	2	1.4	5	2.5	0.704
Differentiation					0.062	MET	1	0.7	5	2.5	0.407
Well	31	21.5	32	15.9		ERBB4	0	0	4	2.0	0.143
Moderate	97	67.4	129	64.2		KIT	0	0	4	2.0	0.143
Poor	16	11.1	40	19.9		ABL1	3	2.1	2	1.0	0.653
ECE ≥ 4			35	17.4		SMO	3	2.1	1	0.5	0.312

CRT, chemoradiotherapy; DBD, DNA-binding domain; ECE, extracapsular extension; HPV, human papilloma virus.

a HPV type 11

b Genes with a mutation rate of ≥ 2% patients in ECE(+) or ECE(−) were included in the analysis.

### Treatment modalities of patients with and without ECE

The distribution of multimodal treatment in patients with and without ECE was as follows: surgery alone, 5% and 13%; surgery plus radiotherapy, 20% and 54%; and surgery plus CRT, 75% and 33%, respectively. Of the 201 patients with ECE, 151 received adjuvant CRT. Most patients (97%; 146/151) received cisplatin-based chemotherapy. Three patients received gemcitabine, while two patients received other drugs. The dosage of cisplatin was 100 mg/m^2^ tri-weekly, 50 mg/m^2^ biweekly [15], or 30-40 mg/m^2^ weekly [16] in 29 (19%), 50 (33%), and 67 (44%) patients, respectively. Eighty-two (56%) patients received an accumulated cisplatin dose of ≥ 200 mg/m^2^ during adjuvant CRT. The radiation dose was ≥ 60 cGy in 147 (97.3%) patients.

### Clinical outcomes in patients with and without ECE

The 5-year clinical outcomes in patients with and without ECE were as follows: LRF rates of 43% and 36% (*P* = 0.041); distant metastases (DM) rates of 39% and 12%, (*P* < 0.001); DSS rates of 47% and 70% (*P* < 0.001); and overall survival (OS) rates of 37% and 58% (*P* < 0.001), respectively.

### Prognostic factors in patients with ECE

Univariate (Supplementary Table S2) and multivariate (Table 2) analyses were used to identify prognostic factors in patients with ECE. In multivariate analysis, adjuvant CRT showed an independent positive impact on all clinical outcomes (LRF, DM, DSS, and OS). Conversely, ≥ 4 ECEs showed a detrimental effect on DM, DSS, and OS (but not LRF). Additionally, both < 5 mm margin and HRAS mutation were adverse prognostic factors for DM and DSS. The presence of pT4 disease and CDKN2A mutations negatively affected DSS and OS. High-risk HPV types (16/18) and other genetic mutations did not show an independent association with clinical outcomes.

**Table 2:** Multivariate analysis^a^ in patients stratified according to the presence of neck lymph node extracapsular extension and use of adjuvant therapy

		Locoregional control	Distant metastasis	Disease specific survival	Overall survival
	n (%)	*P* value, HR (95% CI)	*P* value, HR (95% CI)	*P* value, HR (95% CI)	*P* value, HR (95% CI)
Patients with ECE (n = 201)					
Adjuvant CRT	151 (75.1)	0.000, 0.32 (0.19-0.56)	0.002, 0.42 (0.24-0.73)	0.000, 0.28 (0.17-0.43)	0.000, 0.26 (0.17-0.39)
ECE ≥ 4	35 (17.4)		0.000, 3.11 (1.79-5.39)	0.002, 2.17 (1.34-3.52)	0.000, 2.55 (1.58-4.12)
pT4	79 (39.3)			0.048, 2.22 (1.00-4.90)	0.036, 1.99 (1.04-3.81)
pN2	169 (84.1)	0.008, 3.18 (1.35-7.44)			
Margin < 5 mm	32 (15.9)		0.011, 2.16 (1.19-3.94)	0.013, 1.90 (1.14-3.15)	
Tumor invasion					
Skin	25 (12.4)				0.007, 2.15 (1.22-3.76)
Lymph duct					0.019, 0.53 (0.31-0.90)
Level 4-5 lymph node involvement	23 (11.4)	0.000, 3.59 (1.89-6.83)			
CDKN2A mutations	23 (11.4)			0.036, 1.97 (1.04-3.74)	0.002, 2.29 (1.35-3.87)
HRAS mutations	20 (10.0)		0.000, 3.87 (1.98-7.57)	0.002, 2.60 (1.41-4.81)	

Patients with ECE and adjuvant CRT (n = 151)					
ECE ≥ 4	25 (16.6)		0.000, 3.65 (1.77-6.38)	0.005, 2.26 (1.27-4.03)	0.004, 2.19 (1.29-3.72)
pT4	60 (39.7)	0.019, 1.96 (1.12-3.45)			0.004, 1.88 (1.22-2.91)
Margin < 5 mm	25 (16.8)			0.012, 2.14 (1.18-3.88)	
Differentiation			0.052, 1.90 (0.99-3.63)		
Level 4-5 lymph node involvement	18 (11.9)	0.001, 3.36 (1.67-6.78)			
CDKN2A mutations	19 (12.6)				0.001, 2.62 (1.45-4.72)
HRAS mutations	12 (7.9)		0.001, 3.83 (1.69-8.68)	0.003, 3.11 (1.46-6.62)	

Patients treated with CRT and 1-3 nodes with ECE (n = 126)					
Margin (< 5 mm)	18 (14.3)	0.022, 2.48 (1.13-5.44)		0.011, 2.53 (1.24-5.16)	0.015, 2.21 (1.16-4.20)
Level 4/5 lymph node involvement	11 (8.7)	0.048, 2.49 (1.00-6.16)			
HRAS mutations	11 (8.7)		0.001, 4.33 (1.77-10.60)	0.021, 2.67 (1.15-6.16)	
TP53 DBD missense mutations	63 (50.0)			0.044, 1.89 (1.01-3.52)	0.003, 2.16 (1.30-3.57)

a Genes with a mutation rate of ≥ 2% patients were included in the analysis

CI, confidence interval; CRT, chemoradiotherapy; DBD, DNA-binding domain; ECE, extracapsular extension; HR, hazard ratio.

### Recursive partitioning analysis

**Figure 1:**
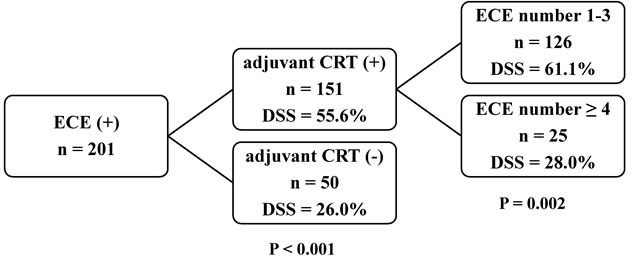
Classification tree derived from recursive partitioning analysis for disease-specific survival Abbreviations: *CRT*, chemoradiotherapy; *DSS*, disease-specific survival; *ECE*, extracapsular extension.

Patients were classified with the use of recursive partitioning analysis (RPA). To this aim, DSS was considered as the dependent variable, whereas independent risk factors identified in multivariate analyses served as predictors/covariates. The classification tree obtained by applying the RPA method identified adjuvant CRT and the number of nodes with ECE as the first and second splitting variables, respectively (Figure 1). The 5-year clinical outcomes of patients who underwent adjuvant CRT *versus* those who did not were as follows: LRF rates of 39% and 58% (*P* = 0.001); DM rates of 33% and 60% (*P* = 0.001); DSS rates of 55% and 21% (*P* < 0.001); and OS rates of 45% and 14% (*P* < 0.001), respectively (Table 3). Patients who were treated with adjuvant CRT were further stratified according to the number of ECEs. The 5-year clinical outcomes of patients with 1-3 *versus* those with ≥ 4 ECEs were as follows: LRF rates of 34% and 67% (*P* = 0.026); DM rates of 28% and 62% (*P* = 0.002); DSS rates of 60% and 25% (*P* = 0.001); and OS rates of 49% and 20% (*P* = 0.005), respectively.

**Table 3:** Clinical outcomes in different patient subgroups

		Locoregional failure		Distant failure		Disease-specific survival		Overall survival	
	n	5-yr rate (%)	Survival^a^ (months)	5-yr rate (%)	Survival (months)	5-yr rate (%)	Survival (months)	5-yr rate (%)	Survival (months)
LN status	345								
ECE (−)	144	36	NR	12	NR	70	NR	58	105
ECE (+)	201	43	NR	39	NR	47	35	37	24
*P* value, HR (95% CI)		0.044, 1,45 (1.01-2.08)		0.000, 3.50 (2.08-5.88)		0.000, 2.31 (1.61-3.30)		0.001, 1.86 (1.39-2.48)	
Presence of ECE	201								
CRTb (+)	151	39	NR	33	NR	55	NR	45	44
CRT (−)	50	58	11	60	14	21	9	14	9
*P* value, HR (95% CI)		0.001, 0.44 (0.27-0.71)		0.001, 0.42 (0.26-0.70)		<0.001, 0.34 (0.23-0.52)		<0.001, 0.37 (0.25-0.53)	
Presence of ECE and history of CRT	151								
ECE 1-3	126	34	NR	28	NR	60	NR	49	70
ECE ≥ 4	25	67	31	62	18	25	14	20	13
*P* value, HR (95% CI)		0.026, 2.10 (1.09-4.02)		0.002, 2.64 (1.41-4.94)		0.001, 2.42 (1.40-4.16)		0.005, 2.04 (1.24-3.37)	
Presence of ECE 1-3 and history of CRT	126								
Margin ≥ 5 mm	108	30	NR			63	NR	52	79
Margin < 5 mm	18	66	33			42	14	33	14
*P* value, HR (95% CI)		0.007, 2.81 (1.32-5.98)				0.039, 2.08 (1.04-4.18)		0.065, 1.79 (0.96-3.34)	
Level 4/5 LN (−)	115	31	NR						
Level 4/5 LN (+)	11	71	8						
*P* value, HR (95% CI)		0.002, 3.62 (1.59-8.23)							
HRAS mutations (−)	115			25	NR	63	NR		
HRAS mutations (+)	11			55	10	36	14		
*P* value, HR (95% CI)				0.007, 3.41 (1.40-8.29)		0.024, 2.52 (1.13-5.62)			
TP53 DBD missense mutations (−)	60					71	NR	61	NR
TP53 DBD missense mutations (+)	63					52	NR	39	42
*P* value, HR (95% CI)						0.025, 1.97 (1.09-3.59)		0.007, 1.97 (1.20-3.22)	

a Median.

CI, confidence interval; CRT, chemoradiotherapy; DBD, DNA-binding domain; ECE, extracapsular extension; HR, hazard ratio; LN, lymph node; NR, not reached; yr, year.

### Multivariate analysis in specific subgroups

**Figure 2:**
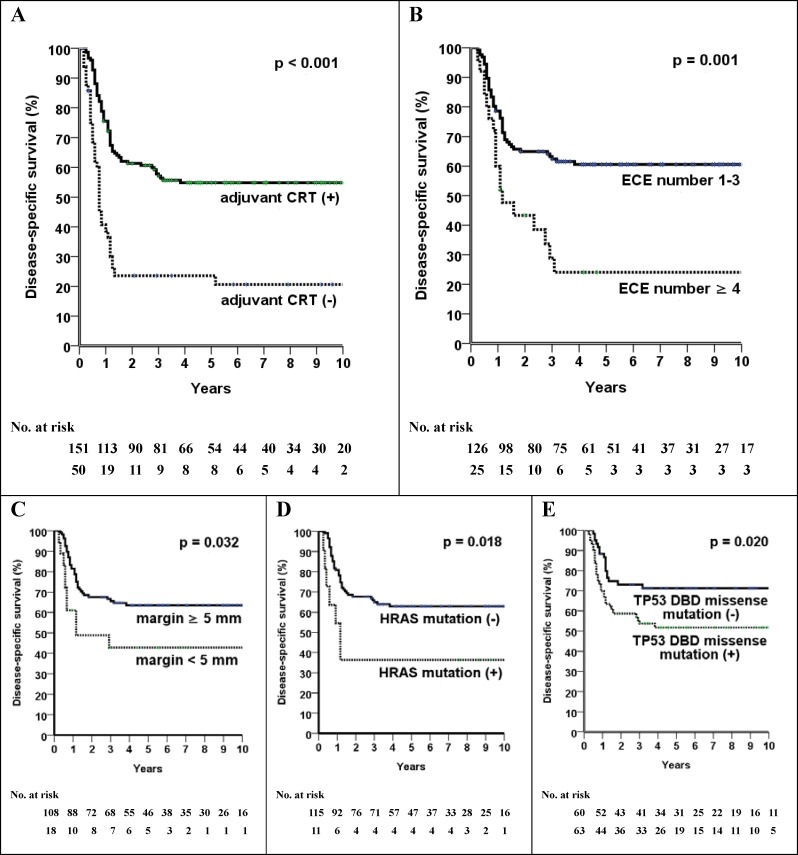
Disease-specific survival stratified according to the use of CRT (**A**), number of nodes with ECE (**B**), and grouping of risk factors in patients treated with CRT for harboring 1-3 nodes with ECE (**C, D, E**). Abbreviations: *CRT*, chemoradiotherapy; *DBD*, DNA-binding domain; *ECE*, extracapsular extension.

In the subgroup of patients who underwent CRT and exhibited 1-3 nodes with ECE (i.e., those with a favorable expected prognosis), we identified the following adverse prognostic factors in multivariate analysis (Table 2): 1) < 5 mm margin for locoregional failure (66% *versus* 30%, *P* = 0.007) and DSS (42% *versus* 63%, *P* = 0.039); 2) HRAS mutations for distant failure (55% *versus* 25%, *P*= 0.007) and DSS (36% *versus* 63%, *P* = 0.024); and 3) TP53 DNA-binding domain missense mutations for DSS (52% *versus* 71%, *P* = 0.025) and OS (39% *versus* 61%, *P* = 0.007). The 5-year DSS and OS rates of patients who had ≥ 4 nodes with ECE were 25% and 20%, respectively. Both, the small sample size of this patient subgroup (*n* = 25) and their dismal outcomes precluded a meaningful multivariate analysis. The clinical outcomes of patients stratified according to the presence of ECE, the use of CRT, and the number of nodes with ECE are shown in Table 3 and Figure 2.

## DISCUSSION

In our study, ECE incidence correlated with worse outcomes in patients with resected OSCC. The 5-year OS rates of patients with and without ECE were 37% and 58%, respectively. However, adjuvant CRT has been reported to improve LRF, DSS, and OS rates, but not that of DM [8–10]. Distant failure occurs more frequently in patients with ECE [4, 17] and buccal cancer [18]. It is noteworthy that the benefit of adjuvant CRT in our high-risk cohort was evident for all time-to-event endpoints, including DM (Table 3). We acknowledge that a comparison of patients who received adjuvant CRT *versus* those who did not should be interpreted cautiously because of selection bias. Indeed, the use of adjuvant CRT in our study was driven by the patient's general conditions and the physician's discretion. The clinical and genomic characteristics of patients with and without CRT were well balanced, the only exception being a higher frequency of patients with a > 10 mm depth of tumor invasion in those who did not receive CRT (65.6% *versus* 88.0%, respectively, *P* = 0.002; Supplementary Table S2). However, the depth of invasion was not an independent predictor of either DSS or DM in multivariate analysis (Table 2). Based on our results, we believe that adjuvant CRT has a positive impact in patients with ECE in terms of all clinical endpoints (i.e., LRF, DSS, OS, and DM). Unfortunately, the outcomes of patients with ECE remain poor even with the use of adjuvant CRT.

Several studies have focused on the prognostic value of ECE extent using different methodological approaches including microscopic *versus* macroscopic ECE [19], a pathological ECE grading system [20], the combination of ECE with other prognostic parameters [6], and the number of nodes showing ECE [4, 21–24]. However, previous reports were heterogeneous in terms of ECE status (positive *versus* negative) [4, 21, 24], primary tumor site (e.g., inclusion of oropharyngeal cancer) [24], and type of adjuvant therapy (RT *versus* CRT) [4, 21, 24]. The presence of more than one node with ECE is the variable most consistently associated with clinical outcomes in previous studies [4, 21, 24]. With RT being the most commonly used adjuvant therapy, a 5-year survival of < 20% has been reported for patients with an ECE number > 1 [4, 21, 24]. In the current study, 151 patients who received adjuvant CRT due to the presence of ECE were included. Their 5-year OS rates stratified according to the number of nodes with ECE ≥ 2, ≥ 3, and ≥ 4 were 44%, 43%, and 20%, respectively. These findings indicate that modern adjuvant CRT may mitigate the adverse prognostic significance of the number of lymph nodes with ECE. With the future goal of implementing precision medicine, it is paramount to provide a molecular refinement of prognosis for patients who have 1-3 nodes with ECE and lack of response to adjuvant CRT. Multivariate analysis (Table 2) revealed the adverse prognostic impact of a < 5 mm margin on LRF and DSS; HRAS mutations on DM and DSS; and TP53 DBD missense mutations on DSS and OSS. Importantly, the outcome of patients without such risk factors was similar to that of subjects without ECE (Table 3).

TP53 is the most commonly mutated gene in HNSCC (60%−80% cases) [13, 25], with its mutations being associated with an unfavorable prognosis [26–28]. Poeta et al. [27] found an association between disruptive TP53 mutations and decreased OS in 420 patients with resected HNSCC enrolled between 1996 and 2002. In their study, 180 patients with OSCC were included, but no data were available regarding their ECE status and adjuvant CRT. The mutations that introduced a stop codon or non-conservative mutations occurring in specific DBDs were defined as disruptive. However, disruptive mutations include two biologically different subtypes, namely 1) truncating mutations associated with a loss of tumor suppressive activity, and 2) DBD missense mutations. DBD missense mutations can result in a gain of function [29] and have been previously associated with decreased DSS in OSCC patients [14]. To the best of our knowledge, this is the first report where the adverse prognostic significance of TP53 DBD missense mutations in patients treated with adjuvant CRT and showed 1-3 nodes with ECE. As discussed above, patients showing 1-3 nodes with ECE are expected to benefit most from adjuvant CRT. Our current observations are in line with previous data reporting an association between TP53 mutations, platinum resistance, and unfavorable outcomes [28, 30]. The impact of TP53 DBD missense mutations on OS (*P* = 0.003) seems greater than that on DSS. However, the percentages of patients with TP53 DBD missense mutations who survived, died of disease-specific causes, and died of competing causes were 27.6%, 57.1%, and 15.3%, respectively. The corresponding figures in patients without TP53 DBD missense mutations were 41.1%, 43.2%, and 15.8%, respectively. Consequently, the observed effect of TP53 DBD missense mutations on OS seems to be driven mainly by disease-specific mortality.

The RAS gene family has been repeatedly shown to be involved in the molecular pathogenesis of OSCC. Specifically, mean mutation rates of 11.2%, 4.5%, and 0.3% have been reported for the HRAS, KRAS, and NRAS genes, respectively [31]. HRAS mutations are common in Asian patients who live in areas where chewing betel quid is an endemic habit [31]. The RAS pathway mediates cellular responses to growth signals and is frequently deregulated in oral cancer. In addition, HRAS mutations have an adverse prognostic impact in terms of PFS and OS rates [32]. In our patients with ECE, the mutation rates of the HRAS, KRAS, and NRAS genes were 10%, 1.5%, and 0%, respectively. Although the locoregional failure rates of patients with and without HRAS mutations were similar (38% and 35%, respectively; *P* = 0.785), the distant failure rate was significantly higher in the former group (65% *versus* 32%, respectively; *P* = 0.003). Differently from HRAS mutations that showed an adverse impact on distant failure, the detrimental effect of < 5 mm margin was in terms of locoregional failure.

In patients with and without CDKN2A mutations, the rates of TP53 DBD missense mutations were 73.7% and 48.3%, respectively (*P* = 0.051). In addition, the frequencies of HRAS mutations were 30.4% and 7.3%, respectively (*P* = 0.000). The coexistence of CDKN2A mutations with other prognostically adverse genetic variations may explain why the significance of the former on DSS and OS (which was present in the cohort of 201 patients harboring ECE) was not replicated when the analysis was based on the number of nodes with ECE.

Patients with ≥ 4 nodes with ECE showed a dismal prognosis despite the implementation of adjuvant therapy. Consequently, the clinical management of high-risk patients (i.e., patients with an ECE number ≥ 4 or 1-3 nodes with ECE and concomitant risk factors) needs to be improved. To achieve this goal, potential future directions include early diagnosis of relapses [33], pre-RT early systemic therapy [34, 35], the combination of different chemotherapy agents with RT [36], the use of altered fractionation/dose escalation RT [37], and genotype-driven targeted therapies [32].

Some caveats of our report merit consideration. First, our study has a retrospective design that makes it prone to selection bias and the presence of potential confounders that were not accounted for. Another potential limitation lies in the identification of the optimal cut-off for the number of nodes with ECE in relation to adverse outcomes. The cut-off of 4 used for this study needs further validation in independent studies. Finally, our observations should be viewed as exploratory and hypothesis generating. Conversely, our study also has significant strengths. Accordingly, this is the largest cohort of homogenously staged, treated, and followed-up OSCC patients with ECE treated with state-of-the-art adjuvant CRT.

In conclusion, our data demonstrate that OSCC is a mutationally heterogeneous malignancy. The combined assessment of the tumor mutation spectrum with traditional clinicopathological risk factors may help refining the prediction of specific clinical outcomes. Further improvements in sequencing techniques and independent validation of our results in distinct cohorts will be necessary to implement precision medicine in resected OSCC patients harboring ECE.

## PATIENTS AND MATERIALS

### Patients

This study includes a subanalysis of a previously described cohort [12] consisting of 345 patients with treatment-naïve, resected OSCC. All participants showed evidence of nodal disease and tumor samples were available (which were analyzed by ultra-deep targeted sequencing as previously described [12]). The subgroup of patients with evidence of ECE (*n* = 201; diagnosed by microscopic visualization of tumor penetration into the lymph node capsule) was the main focus of the study. Ethical approval was granted by the Institutional Review Board of the Chang Gung Memorial Hospital (CGMH 101-4457B). Due to the retrospective nature of the study, the need for patient consent was waived.

### Treatment approach and follow-up schedule

Although all patients with ECE were encouraged to undergo adjuvant CRT, its final use depended on both, patient's willingness and physician's discretion. The most commonly used chemotherapy regimens consisted of cisplatin 100 mg/m^2^ tri-weekly, 50 mg/m^2^ biweekly [15], or 30-40 mg/m^2^ weekly [16]. An adjuvant RT dose of 66 Gy (given in 33 fractions) was generally administered within 6 weeks of surgical resection. After the completion of therapy, patients underwent a follow-up consisting of physical examination every 3 months for the first two years, every 4 to 6 months for the third year, and on an annual basis thereafter. Patients underwent magnetic resonance imaging (MRI)/computed tomography (CT), and/or ^18^F-fluorodeoxyglucose-positron emission tomography (^18^F-FDG-PET) at 3 months after the completion of therapy, and subsequently at 12, 18, and 24 months. Thereafter, symptom-directed imaging was performed. Cases with suspected relapse unscheduled assessments.

### Mutation analysis

The hotspot mutation regions of 45 cancer-related genes were examined using NGS with an ultra-deep (>1000×) sequencing approach in FFPE, primary tumor samples as previously described [12]. We examined the following 29 oncogenes and 16 tumor suppressor genes (TSG): ABL1, AKT1, ALK, APC, ATM, BRAF, CDH1, CDKN2A, CSF1R, CTNNB1, EGFR, ERBB2, ERBB4, FBXW7, FGFR1, FGFR2, FGFR3, FLT3, GNAS, HNF1A, HRAS, IDH1, JAK3, KDR, KIT, KRAS, MET, MLH1, MPL, NOTCH1, NPM1, NRAS, PDGFRA, PIK3CA, PTEN, PTPN11, RB1, RET, SMAD4, SMARCB1, SMO, SRC, STK11, TP53, and VHL. In a separate study, we examined the value of missense mutations affecting the TP53 DBD (but not of the remaining TP53 mutations) for the prediction of DSS [14]. HPV infections were diagnosed using HPV L1 gene PCR. In patients who tested positive, the HPV L1 gene was genotyped using an HPV Blot kit (EasyChipTM, King Car Ltd., Yilan, Taiwan) that can differentiate between 39 different HPV types [38].

### Study endpoints

The study endpoints included LRF, DM, DSS, and OS. All time-to-event endpoints were calculated from the date of surgery to the date of the event of interest (or censored on the date of the last follow-up).

### Data analysis

Categorical data were compared using Fisher's exact test (2 × 2 tables). Continuous variables were examined with independent-sample Student's *t*-test. Univariate analysis was performed by logistic regression. Kaplan-Meier plots (log-rank tests) were used to summarize the course of time-to-event data. Multivariate Cox proportional hazards models were used to identify the independent predictors of clinical outcomes. Recursive partitioning analysis (RPA) was then used to classify patients into successively more homogeneous prognostic groups based on multiple input variables. All calculations were performed using the SPSS statistical package, version 18.0 (SPSS Inc., Chicago, IL, USA). *P* values < 0.05 (two-tailed) were considered statistically significant.

## SUPPLEMENTARY TABLES


