# Supplementary material for: Clues toward precision medicine in oral squamous cell carcinoma: utility of next-generation sequencing for the prognostic stratification of high-risk patients harboring neck lymph node extracapsular extension
Source: Oncotarget. 2016 Aug 31;7(39):63082–92. doi: 10.18632/oncotarget.11762 (PMC5325348; doi:10.18632/oncotarget.11762)
Supplement: Supplementary file 1 [file oncotarget-07-63082-s001.docx]

**Supplementary Table S1. Rare mutated genes^a^ of the study patients stratified according to the presence of neck lymph node extracapsular extension**

|  | ECE (-) | | ECE (+) | | |  | |
| --- | --- | --- | --- | --- | --- | --- | --- |
|  | n = 144 | | n = 201 | | |  | |
| Genetic mutations | n | % | | n | % | | P value |
| KRAS3 | 2 | 1.4 | | 3 | 1.5 | | 1.000 |
| APC | 0 | 0 | | 3 | 1.5 | | 0.268 |
| CDH1 | 0 | 0 | | 3 | 1.5 | | 0.268 |
| RB1 | 0 | 0 | | 3 | 1.5 | | 0.268 |
| SMARCB1 | 0 | 0 | | 3 | 1.5 | | 0.268 |
| STK11 | 0 | 0 | | 3 | 1.5 | | 0.268 |
| ERBB2 | 2 | 1.4 | | 2 | 1.0 | | 1.000 |
| FBXW7 | 1 | 0.7 | | 2 | 1.0 | | 1.000 |
| NOTCH1 | 1 | 0.7 | | 2 | 1.0 | | 1.000 |
| RET | 1 | 0.7 | | 2 | 1.0 | | 1.000 |
| FGFR1 | 0 | 0 | | 2 | 1.0 | | 0.512 |
| PTPN11 | 0 | 0 | | 2 | 1.0 | | 0.512 |
| ATM | 2 | 1.4 | | 1 | 0.5 | | 0.573 |
| AKT1 | 0 | 0 | | 1 | 0.5 | | 1.000 |
| CTNNB1 | 0 | 0 | | 1 | 0.5 | | 1.000 |
| PDGFRA | 1 | 0.7 | | 1 | 0.5 | | 1.000 |
| FGFR2 | 0 | 0 | | 1 | 0.5 | | 1.000 |
| FLT3 | 0 | 0 | | 1 | 0.5 | | 1.000 |
| IDH1 | 0 | 0 | | 1 | 0.5 | | 1.000 |
| MLH1 | 0 | 0 | | 1 | 0.5 | | 1.000 |
| NPM1 | 0 | 0 | | 1 | 0.5 | | 1.000 |
| PTEN | 0 | 0 | | 1 | 0.5 | | 1.000 |
| VHL | 0 | 0 | | 1 | 0.5 | | 1.000 |
| NRAS | 1 | 0.7 | | 0 | 0 | | 0.417 |
| SRC | 1 | 0.7 | | 0 | 0 | | 0.417 |

^a^ Genes with a mutation rate of < 2% patients in both, ECE(+) and ECE(-) groups.

**Supplementary Table S2. Univariate analysis in patients with neck lymph node extracapsular extension^a^ (n = 201)**

|  |  | **Locoregional control** | **Distant metastasis** | **Disease-specific survival** | **Overall survival** |
| --- | --- | --- | --- | --- | --- |
|  | **n (%)** | **P value, HR (95% CI)** | **P value, HR (95% CI)** | **P value, HR (95% CI)** | **P value, HR (95% CI)** |
| Male sex (vs female) | 191(95) | 0.249, 2.53 (0,52-12.24) | 0.751, 0.81 (0.22-2.97) | 0.450, 1.64 (0.45-6.02) | 0.384, 0.49 (0.10-2.40) |
| Age: >70 years (vs ≤ 70 years) | 8(4.0) | 0.452, 0.53 (0.10-2.72) | 0.895, 1.10 (0.25-4.75) | 0.920, 0.93 (0.22-3.82) | 0.240, 3.55 (0.42-29.51) |
| Cancer site |  |  |  |  |  |
| ICD-9-CM, 145 (vs not 145) | 90 (44.8) | 0.553, 0.84 (0.47-1.49) | 0.212, 1.44 (0.80-2.58) | 0.209, 1.43 (0.81-2.50) | 0.662, 0.87 (0.48-1.58) |
| pT3-4 (vs pT1-2) | 122 (60.7) | 0.578, 1.18 (0.65-2.12) | 0.018, 2.11 (1.13-3.94) * | 0.002, 2.51 (1.40-4.50) * | 0.001, 2.82 (1.53-5.18) * |
| pT4 (vs pT1-3) | 79 (39.3) | 0.352, 1.31 (0.73-2.35) | 0.125, 1.58 (0.88-2.85) | 0.078, 1.67 (0.94-2.97) | 0.034, 1.97 (1.05-3.72) * |
| pN2 (vs N1) | 169 (84.1) | 0.019, 3.06 (1.19-7.83) * | 0.088, 2.17 (0.89-5.32) | 0.035, 2.33 (1.06-5.14) * | 0.840, 1.08 (0.48-2.41) |
| Stage IV (vs III) | 179 (89.1) | 0.130, 2.23 (0.78-6.33) | 0.084, 2.69 (0.87-8.29) | 0.053, 2.53 (0.98-6.51) | 0.186, 1.83 (0.74-4.48) |
| Differentiation |  |  |  |  |  |
| Poor (vs well and moderate) | 40 (19.9) | 0.682, 0.86 (0.41-1.77) | 0.032, 2.15 (1.06-4.35) * | 0.245, 1.51 (0.75-3.07) | 0.960, 1.01 (0.48-2.13) |
| ECE number ≥ 2 | 107 (53.2) | 0.107, 1.60 (0.90-2.86) | 0.344, 1.32 (0.74-2.37) | 0.456, 1.23 (0.70-2.15) | 0.575, 0.84 (0.46-1.52) |
| ECE number ≥ 3 | 59 (29.4) | 0.391, 1.31 (0.70-2.43) | 0.047, 1.87 (1.00-3.50) * | 0.167, 1.54 (0.83-2.85) | 0.434, 1.30 (0.67-2.52) |
| ECE number ≥ 4 | 35 (17.4) | 0.071, 1.97 (0.95-4.11) | 0.001, 3.48 (1.63-7.39) * | 0.002, 3.90 (1.67-9.08) * | 0.035, 2.73 (1.07-6.96) * |
| Margin < 5 mm (vs ≥ 5 mm) | 32 (15.9) | 0.114, 1.84 (0.86-3.95) | 0.056, 2.11 (0.98-4.53) | 0.037, 2.36 (1.05-5.29) * | 0.297, 1.58 (0.66-3.74) |
| Depth of invasion > 10 mm | 143 (71.1) | 0.982, 0.99 (0.52-1.86) | 0.016, 2.36 (1.17-4.76) * | 0.002, 2.70 (1.43-5.10) * | 0.009, 2.32 (1.23-4.37) * |
| Invasion: Bone | 44 (21.9) | 0.898, 1.04 (0.52-2.08) | 0.603, 1.20 (0.60-2.39) | 0.674, 1.15 (0.59-2.25) | 0.110, 1.88 (0.86-4.10) |
| Skin | 25(12.4) | 0.809, 1.11 (0.47-2.61) | 0.334, 1.51 (0.65-3.55) | 0.035, 2.69 (1.07-6.76) * | 0.065, 2.85 (0.93-8.69) |
| Nerve | 113(56.2) | 0.167, 0.66 (0.37-1.18) | 0.132, 1.57 (0.87-2.85) | 0.663, 1.13 (0.64-1.97) | 0.786, 0.92 (0.50-1.66) |
| Blood vessel | 12(6.0) | 0.777, 1.18 (0.36-3.88) | 0.882, 0.91 (0.26-3.13) | 0.901, 0.92 (0.28-2.98) | 0.504, 0.667 (0.20-2.18) |
| Lymphatic duct | 33(16.4) | 0.851, 0.92 (0.42-2.01) | 0.593, 1.23 (0.57-2.65) | 0.464, 1.32 (0.62-2.81) | 0.458, 1.36 (0.59-3.14) |
| Level 4/5 lymph node involvement | 23(11.4) | 0.019, 2.91 (1.19-7.10) * | 0.078, 2.20 (.091-5.28) | 0.075, 2.33 (0.91-5.95) | 0.466, 1.44 (0.54-3.84) |
| Adjuvant CRT^b^ | 151(75.1) | 0.088, 0.56 (0.29-1.08) | 0.032, 0.49 (0.25-0.94) * | 0.000, 0.28 (0.13-0.56) * | 0.001, 0.20 (0.08-0.51) * |
| cDDP total ≥ 200 mg/m^2^ | 82 (54.3)^a^ | 0.442, 1.30 (0.66-2.55) | 0.591, 1.20 (0.60-2.41) | 0.267, 1.44 (0.75-2.75) | 0.071, 1.84 (0.94-3.59) |
| HPV (type 16, 18 vs others) | 27(13.4) | 0.359, 1.47 (0.64-3.36) | 0.615, 0.79 (0.32-1.93) | 0.883, 1.06 (0.47-2.40) | 1.000, 1.00 (0.42-2.37) |
| **Genetic mutations^b^** |  |  |  |  |  |
| TP53 CPV2 | 123(61.2) | 0.883, 1.04 (0.58-1.87) | 0.010, 2.27 (1.21-4.26) * | 0.066, 1.70 (0.96-3.03) | 0.050, 1.82 (1.00-3.31) |
| TP53 DBD missense mutations ^c^ | 98(48.8) | 0.777, 1.08 (0.60-1.95) | 0.011, 2.21 (1.20-4.09) * | 0.053, 1.75 (0.99-3.10) | 0.049, 1.83 (1.00-3.34) * |
| CDKN2A | 23(11.4) | 0.552, 1.30 (0.54-3.14) | 0.187, 1.80 (.075-4.32) | 0.075, 2.33 (0.91-5.95) | 0.019, 5.89 (1.33-25.95) * |
| PIK3CA | 23(11.4) | 0.750, 0.86 (0.34-2.14) | 0.603, 0.77 (0.30-1.99) | 0.401, 0.68 (0.28-1.65) | 0.795, 1.13 (0.44-2.90) |
| HRAS | 20(10.0) | 0.785, 0.87 (0.33-2.29) | 0.006, 3.93 (1.49-10.39) * | 0.013, 4.22 (1.36-13.13) * | 0.086, 3.02 (0.85-10.71) |
| BRAF | 7(3.5) | 0.295, 2.25 (0.49-10.38) | 0.705, 0.72 (0.13-3.83) | 0.771, 1.25 (0.27-5.74) | 0.807, 1.23 (0.23-6.51) |
| EGFR | 6(3.0) | 0.162, 3.14 (0.61-19.12) | 0.918, 0.91 (0.16-5.11) | 0.465, 1.90 (0.34-10.61) | 0.999, 8.265E8 (0.00-.) |
| FGFR3 | 6(3.0) | 0.819, 0.81 (0.14-4.57) | 0.452, 1.86 (0.36-9.50) | 0.465, 1.90 (0.34-10.61) | 0.999, 8.265E8 (0.00-.) |
| SMAD4 | 6(3.0) | 0.162, 3.41 (0.61-19.12) | 0.452, 1.88 (0.36-9.50) | 0.153, 4.84 (0.55-42.26) | 0.999, 8.265E8 (0.00-.) |
| KDR | 5(2.5) | 0.807, 6.88 (0.75-62.82) | 0.825, 1.22 (0.20-7.52) | 0.233, 3.84 (0.42-34.97) | 0.999, 8.202E8 (0.00-.) |
| MET | 5(2.5) | 0.421, 0.40 (0.04-3.67) | 0.070, 7.70 (0.84-70.28) | 0.233 ,3.84 (0.42-34.97) | 0.999, 8.202E8 (0.00-.) |
| ERBB4 | 4(2.0) | 0.615, 1.66 (0.22-12.05) | 0.541, 1.85 (0.25-13.45) | 0.944, 0.93 (0.12-6.74) | 0.738, 1.47 (0.15-14.48) |
| KIT | 4(2.0) | 0.615, 1.66 (0.22-12.05) | 0.666, 0.60 (0.06-5.92) | 0.944, 0.93 (0.12-6.74) | 0.738, 1.47 (0.15-14.48) |

***cDDP****,* cisplatin; ***CI***, confidence interval; ***CRT***, chemoradiotherapy; ***DBD***, DNA-binding domain; ***HPV***, human papillomavirus; ***HR***, hazard ratio.

***ICD-9-CM 145***, International Classification of Disease, 9th version, Clinical Modification, code 145

^a^ A total of 151 patients received CRT; ^b^ Genes with a mutation rate of ≥ 2% patients were included in the analysis; ^c^ Data from 193 patients.

* P < 0.05

**Supplementary Table S3. Characteristics of patients with neck lymph node extracapsular extension (n = 201) stratified according to the use of chemoradiotherapy**

|  | CRT (+) | CRT (-) |  |  | CRT (+) | CRT (-) |  |
| --- | --- | --- | --- | --- | --- | --- | --- |
|  | N = 151 | N = 50 |  |  | N = 151 | N = 50 |  |
| Variables | % | % | P value |  | % | % | P value |
| Sex |  |  | 0.712 | Presence of mutations | 72.2 | 78.0 | 0.419 |
| Male | 95.4 | 94.0 |  | Number of mutations (mean) | 1.30 ± 2.08 | 1.58 ± 3.04 | 0.475 |
| Female | 4.6 | 6.0 |  | TP53 | 60.9 | 62.0 | 0.883 |
| Age, years |  |  | 0.063 | TP53 DBD missense mutations (n = 193) | 51.4 | 48.9 | 0.772 |
| Mean | 48.9 ± 10.7 | 52.4 ± 12.8 |  |  |  |  |  |
| Age >70 years | 3.3 | 6.0 | 0.413 | HPV (n = 187) |  |  | 0.367 |
| Cancer site |  |  | 0.149 | None | 83.0 | 91.3 |  |
| Tongue | 37.1 | 36.0 |  | Type 16, 18 | 16.3 | 8.7 |  |
| Mouth floor | 2.6 | 10.0 |  | Other HPV type ^a^ | 0.7 | 0 |  |
| Lip | 0.7 | 0 |  | CDKN2A | 12.6 | 8.0 | 0.452 |
| Buccal | 41.1 | 36.0 |  | PIK3CA | 11.9 | 10.0 | 0.803 |
| Gum | 11.9 | 18.0 |  | HRAS | 7.9 | 16.0 | 0.108 |
| Hard palate | 0.7 | 0 |  | BRAF | 2.0 | 8.0 | 0.066 |
| Retromolar | 6.0 | 0 |  | EGFR | 2.6 | 4.0 | 0.640 |
| Tumor status (1) |  |  | 0.068 | FGFR3 | 3.3 | 2.0 | 1.000 |
| 1 | 3.3 | 2.0 |  | SMAD4 | 2.0 | 6.0 | 0.164 |
| 2 | 39.7 | 26.0 |  | KDR | 2.6 | 2.0 | 1.000 |
| 3 | 17.2 | 34.0 |  | MET | 1.3 | 6.0 | 0.100 |
| 4 | 39.7 | 38.0 |  | ERBB4 | 1.3 | 4.0 | 0.259 |
| Lymph node status |  |  | 0.507 | KIT | 2.0 | 2.0 | 1.000 |
| N1 | 14.6 | 20.0 |  | KRAS3 | 0.7 | 4.0 | 0.153 |
| N2a | 1.3 | 2.0 |  | APC | 1.3 | 2.0 | 1.000 |
| N2b | 71.5 | 60.0 |  | CDH1 | 1.3 | 2.0 | 1.000 |
| N2c | 12.6 | 18.0 |  | RB1 | 1.3 | 2.0 | 1.000 |
| Stage |  |  | 0.438 | SMARCB1 | 2-0 | 0 | 0.575 |
| III | 9.9 | 14.0 |  | STK11 | 1.3 | 2.0 | 1.000 |
| IV | 90.1 | 86.0 |  | ABL1 | 1.3 | 0 | 1.000 |
| Differentiation |  |  | 0.775 | ERBB2 | 1.3 | 0 | 1.000 |
| Well | 15.2 | 18.0 |  | FBXW7 | 0.7 | 2.0 | 0.437 |
| Moderate | 65.6 | 60.0 |  | NOTCH1 | 0 | 4.0 | 0.061 |
| Poor | 19.2 | 22.0 |  | RET | 1.3 | 0 | 1.000 |
| ECE number ≥ 4 | 16.6 | 20.0 | 0.578 | FGFR1 | 0.7 | 2.0 | 0.437 |
| Margin < 5 mm | 16.6 | 14.9 | 1.000 | PTPN11 | 1.3 | 0 | 1.000 |
| Depth of invasion > 10 mm | 65.6 | 88.0 | 0.002 | SMO | 0.7 | 0 | 1.000 |
| Tumor invasion |  |  |  | ATM | 0 | 2.0 | 0.249 |
| Bone | 20.5 | 26.0 | 0.417 | AKT1 | 0 | 2.0 | 0.249 |
| Skin | 11.9 | 14.0 | 0.805 | CTNNB1 | 0.7 | 0 | 1.000 |
| Nerve | 59.6 | 46.04 | 0.093 | PDGFRA | 0.7 | 0 | 1.000 |
| Blood vessel | 6.0 | 6.0 | 1.000 | FGFR2 | 0 | 2.0 | 0.249 |
| Lymphatic duct | 15.2 | 20.0 | 0.430 | FLT3 | 0.7 | 0 | 1.000 |
| Level 4/5 lymph nodes | 11.9 | 10.0 | 0.803 | IDH1 | 0.7 | 0 | 1.000 |
|  |  |  |  | MLH1 | 0.7 | 0 | 1.000 |
|  |  |  |  | NPM1 | 0 | 2.0 | 0.249 |
|  |  |  |  | PTEN | 0.7 | 0 | 1.000 |
|  |  |  |  | VHL | 0.7 | 0 | 1.000 |
|  |  |  |  | NRAS | 0 | 0 | NA |
|  |  |  |  | SRC | 0 | 0 | NA |

***CRT***, chemoradiotherapy; ***DBD***, DNA-binding domain; ***ECE***, extracapsular extension; ***HPV***, human papillomavirus; **NA**, not available.
